# Cadence Modulation in Walking and Running: Pacing Steps or Strides?

**DOI:** 10.3390/brainsci10050273

**Published:** 2020-05-01

**Authors:** Anouk Nijs, Melvyn Roerdink, Peter J. Beek

**Affiliations:** Faculty of Behavioural and Movement Sciences, Vrije Universiteit Amsterdam, Amsterdam Movement Sciences, van der Boechorststraat 7-9, 1081 BT Amsterdam, The Netherlands

**Keywords:** acoustic pacing, cadence, perturbations, relative phase, sensorimotor synchronization, coordinative stability

## Abstract

A change in cadence during walking or running might be indicated for a variety of reasons, among which mobility improvement and injury prevention. In a within-subject study design, we examined whether walking or running cadences are modulated best by means of step-based or stride-based auditory pacing. Sixteen experienced runners walked and ran on a treadmill while synchronizing with step-based and stride-based pacing at slow, preferred and fast pacing frequencies in synchronization-perturbation and synchronization-continuation conditions. We quantified the variability of the relative phase between pacing cues and footfalls and the responses to perturbations in the pacing signal as measures of coordinative stability; the more stable the auditory-motor coordination, the stronger the modulating effect of pacing. Furthermore, we quantified the deviation from the prescribed cadence after removal of the pacing signal as a measure of internalization of this cadence. Synchronization was achieved less often in running, especially at slow pacing frequencies. If synchronization was achieved, coordinative stability was similar, and the paced cadence was well internalized for preferred and fast pacing frequencies. Step-based pacing led to more stable auditory-motor coordination than stride-based pacing in both walking and running. We therefore concluded that step-based auditory pacing deserves preference as a means to modulate cadence in walking and running.

## 1. Introduction

Increasing cadence in locomotion might be beneficial for a variety of reasons and auditory pacing could well be used to achieve this. For instance, with respect to walking it has been suggested that increasing cadence of stroke patients, who often have a reduced ability to modulate cadence, could help increase their walking speed and improve mobility [1,2]. Most stroke patients are able to adjust their cadence to an external beat [3,4,5]. With regard to running, research has suggested that running injuries due to overload might be reduced by increasing cadence at a given speed [6,7]. Small increases in cadence (+ 10%, and corresponding decreases in step length [8]) result in a reduction of energy absorption in the joints of the lower extremities [7] as well as a decrease in braking impulse and instantaneous vertical loading rate [9]. Runners are also able to couple their cadence to an external beat [10,11].

Auditory pacing is commonly used to prescribe a particular movement frequency in cyclic tasks, including locomotion. In locomotion, auditory pacing can be readily provided via a headset. Auditory cues in the form of a beat can be used to influence the movement pattern through a process known as auditory-motor synchronization [11,12,13,14,15,16,17], defined here as the coordination of rhythmic movements, such as steps, to external stimuli, such as metronome beeps. Stable coordination between steps and cues is associated with a strong effect of pacing on the movement pattern [4,18]. The stability of auditory-motor coordination is often examined in terms of the variability of the relative phase between steps and cues, with lower variability representing higher stability [4,13,19]. In addition, step adjustments to rhythm perturbations in the pacing signal can be used to assess the stability of auditory-motor coordination, with faster corrections representing higher stability [4,12,20,21].

Two factors influence auditory-motor coordination: coupling strength and frequency detuning [22]. Coupling strength refers to how weak or strong the interaction is between two oscillators, here between rhythmic limb movements and auditory cues. Different types of pacing (e.g., one or two cues per movement cycle) are associated with different coupling strengths, which affect the stability of auditory-motor coordination [22]. Most research on auditory pacing has been conducted in the context of finger and hand movements [12,23,24,25], but the same concept holds for auditory-motor coordination during locomotion [13,14,26]. In finger and hand movement tasks, coupling is stronger for one cue per tap (1:1 ratio) than for one cue every other tap (1:2 ratio) [22], resulting in superior auditory-motor coordination. One study compared a 1:1 ratio (pacing steps) to a 1:2 ratio (pacing strides) in walking [4]. As for tapping, a superior auditory-motor coordination for the 1:1 ratio (one cue per step; stronger coupling) was found compared to the 1:2 ratio (one cue per stride, that is, one cue every other step; weaker coupling). In the present study, we sought to verify these coupling-strength effects for walking and to extend them to running using a within-subjects design requiring experienced runners (i.e., participants who could perform all conditions).

In tapping, synchronization between taps and cues typically occurs within a certain frequency range [12,27]. That is, if the difference between the preferred unpaced tapping frequency and the imposed pacing frequency becomes too large, synchronization becomes impossible. This relates to the second factor affecting auditory-motor coordination, frequency detuning, defined for two coupled oscillators as the mismatch between their intrinsic frequencies [22,28]. Modulating cadence with auditory pacing by definition implies detuning, as pacing frequencies differ from one’s preferred cadence. With a frequency mismatch, synchronization between steps and cues may or may not occur, depending on the strength of the coupling (i.e., weaker for pacing strides than for pacing steps) and the magnitude of the mismatch (i.e., how much the paced frequency differs from one’s preferred cadence in the absence of pacing). Hence, fewer occurrences of synchronization are to be expected for stride-based pacing (weaker coupling) than for step-based pacing (stronger coupling), and particularly so for pacing frequencies other than one’s preferred frequency. If synchronization does occur, detuning is expected to affect the stability of auditory-motor coordination [13,22], with superior auditory-motor coordination (i.e., lower relative-phase variability, faster responses to rhythm perturbations) at one’s preferred cadence (i.e., no detuning). Detuning also affects lead-lag relationships between steps and pacing cues during synchronized auditory-motor coordination [12,22]. Footfalls typically precede pacing cues (i.e., anticipation tendency [12,24]), with the magnitude of this phase lead depending on the frequency mismatch between the pacing frequency and one’s preferred cadence in the absence of pacing [12,13]. In line with this detuning effect, the intrinsically faster oscillator (i.e., walker/runner paced at slower-than-preferred cadence) tends to lead and the intrinsically slower oscillator (i.e., walker/runner paced at faster-than-preferred cadence) tends to lag the anticipation tendency seen in the absence of detuning (i.e., walker/runner paced at their preferred cadence) [12,13]. The mean relative phase between cues and steps indicates the phase lead/lag.

Not only do indicators of synchronization and the stability of auditory-motor coordination matter when one wants to modulate cadence with auditory pacing, but also the extent to which the prescribed cadence can be internalized when pacing is turned off. This modulating effect of pacing in terms of internalization of the prescribed cadence can be examined using a synchronization-continuation paradigm, with lower deviations from the prescribed cadence after removal of the pacing signal representing stronger internalization [23,29,30,31]. Furthermore, besides objective findings, the subjective experience of the user about the various pacing types matter for practical reasons such as compliance [32]. Ideally, objective synchronization, stability and internalization findings match participants’ subjective experiences with the various types of cues.

In sum, the purpose of the present study was to compare the effectiveness of step-based and stride-based pacing for modulating walking and running cadences. We expected more synchronization and more stable auditory-motor coordination with step-based pacing (stronger coupling) for both walking and running, reflecting a superior cadence-modulating effect [4,18]. As a reflection of this, we expected participants’ subjective experiences about the various types of cues to match objective synchronization, auditory-motor coordination and internalization findings in that participants would best rate the pacing type with the strongest coupling (i.e., step-based pacing). Furthermore, we expected more stable auditory-motor coordination with congruent pacing (i.e., pacing matching one’s preferred cadence), and decreasing phase leads with faster pacing. Finally, we expected a larger detuning-related deviation from the prescribed cadence after removal of the pacing signal in slow and fast pacing conditions, with a change of cadence towards one’s preferred cadence.

## 2. Materials and Methods

### 2.1. Experiment

#### 2.1.1. Participants

Sixteen healthy runners (6 male/10 female), 29 ± 6 years of age (mean ± standard deviation) participated in the study. Runners were only included if they were uninjured, fell within the age range of 18 to 40 years, ran more than 7.5 km per week, and participated in at least one competitive race in 2018 and 2019. On average, the runners included in the present study ran 29.4 ± 12.7 km per week and participated in 11 ± 7 races in 2018 and 2019.

#### 2.1.2. Ethics

All participants provided written informed consent before participation. The protocol was in accordance with the Declaration of Helsinki and approved by the Scientific and Ethical Review Board (VCWE) of the Faculty of Behavioural and Movement Sciences of the Vrije Universiteit Amsterdam (VCWE-2019-006R1).

#### 2.1.3. Equipment

The participants walked and ran on an instrumented treadmill (Dual-belt; Motekforce Link, Amsterdam), which was equipped with two force platforms. Auditory cues were generated by a computer and provided through speakers. Auditory cues and force-platform data were sampled simultaneously at 500 Hz.

#### 2.1.4. Procedure

All participants completed the measurement protocol for both walking and running (the order of which was counterbalanced over participants; Table 1). Before the measurements started, the participants walked or ran (depending on the upcoming locomotion condition) on the treadmill for approximately 10 min at varying speeds in order to familiarize themselves with the treadmill. At the end of the familiarization period, the speed was increased (and then decreased) and the participants were asked to indicate when the speed was too high (low) to walk/run comfortably for half an hour. These two limits were averaged to obtain a speed that the participants indicated to be comfortable with. If not, the speed was adjusted by 0.5 km/h. When a comfortable speed was established, the participants walked/ran at the determined speed for one minute to determine the preferred cadence.

**Figure 1:**
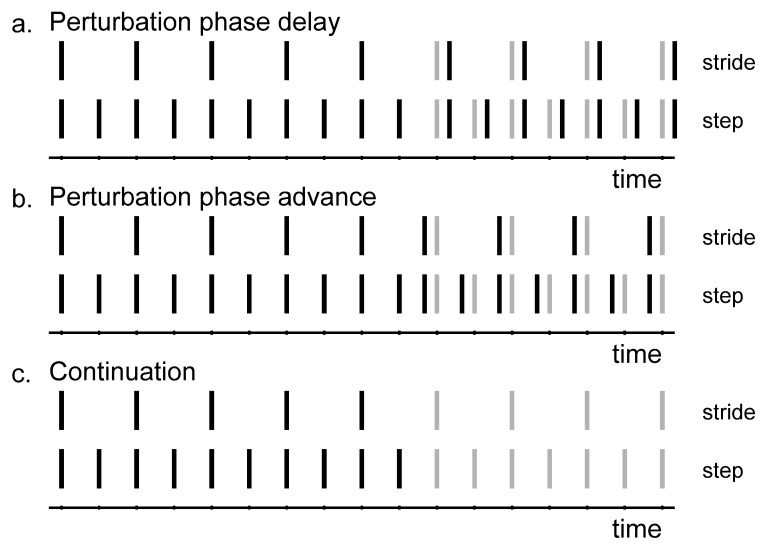
The synchronization-perturbation and synchronization-continuation tasks. Stride-based (upper) and step-based (lower) pacing rhythms are indicated by the vertical lines. (**a**) Phase-delay perturbations. The actual beep (black) comes later than expected according to the rhythm (grey). (**b**) Phase-advance perturbations. The actual beep (black) comes earlier than expected according to the rhythm (grey). (**c**) Continuation. The beeps no longer appear.

In each locomotion block, participants performed two tasks (Table 1): a synchronization-perturbation task (Figure 1a,b; [4,20,21]) and a synchronization-continuation task (Figure 1c; [33,34]). During the synchronization-perturbation task, the participants were paced at a certain cadence. The auditory cues were provided to the participants as a sequence of metronome beeps. Beep duration was 0.05 s, including 0.005 s fading at the start and end of each beep to make them sound less mechanical. After one minute of adaptation and synchronization, 60° phase perturbations were introduced in the pacing signal (360° corresponds to one stride). There were four phase-delay (+60°; Figure 1a) and four phase-advance perturbations (−60°; Figure 1b), separated by 30 to 40 steps. The number of steps between perturbations and the perturbation direction were randomized to ensure that participants could not anticipate the perturbations. Participants were instructed to adjust their footfalls to the cues [20,26]. During the synchronization-continuation task, they were also paced at a certain cadence for one minute. Subsequently, pacing was removed for the final 90 s. Participants were instructed to maintain the imposed cadence (Figure 1c).

Step-based and stride-based pacing were compared for both walking and running (Table 1). For step-based pacing, beeps alternated in pitch (500 Hz and 800 Hz) to pace the footfalls of both feet. For stride-based pacing, beeps of 800 Hz pitch were used to pace one of the two footfalls per gait cycle. All conditions were performed with pacing at the preferred cadence, a slower cadence (0.9 * preferred) and a faster cadence (1.1 * preferred; Table 1).

After all conditions were completed, the participants walked/ran for one minute at the comfortable speed without any cues to verify the preferred cadence. Subsequently, they filled out a questionnaire about how they experienced the types of pacing; in particular, they were asked to rate difficulty, perceived performance, comfort and enjoyment on a visual scale.

Participants completed all 24 conditions (repeated-measures design). The order of both locomotion type and pacing type was counterbalanced over participants. The task and pacing frequency conditions were grouped per locomotion and pacing type condition to avoid confusion for the participants. The order of the pacing frequencies was randomized within each condition (Table 1).

### 2.2. Analysis

MATLAB^®^ (MathWorks^®^, R2018b) was used for all calculations prior to statistical analysis. The dataset, comprising preprocessed series, calculated series and event streams per participant and condition, is available in the Supplementary Materials of this paper.

**Figure 2:**
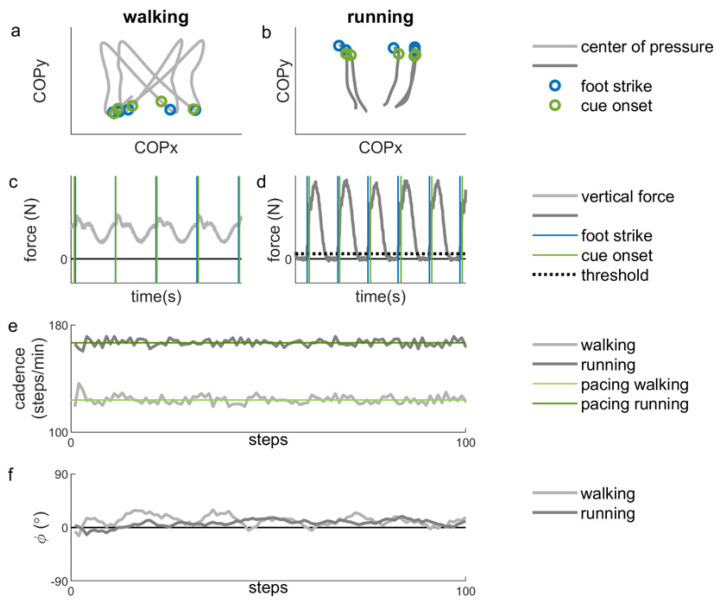
Example of representative data during stationary auditory-motor coordination. (**a**) The filtered (low-pass, second-order Butterworth filter with a cut-off frequency of 10 Hz) center of pressure (COP) for two seconds of walking for one participant. The blue circles indicate instants of foot strike and the green circles indicate cue onsets. (**b**) The filtered center of pressure (COP) for two seconds of running for one participant. The blue circles indicate instants of foot strike and the green circles indicate cue onsets (corresponding to panel d). (**c**) The vertical force data for the same two seconds of walking. The vertical blue lines indicate instants of foot strike and the vertical green lines indicate cue onsets (corresponding to panel a). (**d**) The vertical force data for the same two seconds of running. The vertical blue lines indicate instants of foot strike, defined as the moment in time when the vertical force crosses the threshold of 5% of the maximum vertical force (black dotted line). The vertical green lines indicate cue onsets. (**e**) The cadence for 100 steps of walking (light grey line) and running (dark grey line) and the corresponding (preferred) pacing frequencies (light green line for walking and dark green line for running) (**f**) The relative phase (*ϕ*) for 100 steps of walking (lighter grey line) and running (darker grey line). A positive relative phase indicates that the foot strikes are ahead of the cues.

Cadence was determined based on the moments of foot strike (Figure 2e). For walking, moments of foot strike were extracted from the collected center-of-pressure data (COP; Figure 2a) [35]. For running, foot strike was determined as the moment the vertical component of the force vector reached a preset threshold of 5% of the maximum vertical force (Figure 2d). Cadences were normalized to the preferred cadence as assessed at the start of each locomotion type block.

#### 2.2.1. Synchronization Phase

Per condition, a series of point estimates of relative phase (*ϕ* in °) between the foot strikes and the cues was determined according to:

(1) $$\phi =360{}^{\circ}*\frac{\left(cue\;onset\;-foot\;strike\right)}{interval\;ipsilateral\;cues}$$

where *cue onset* and *foot strike* represent the time instants corresponding, respectively, to the cue onsets and the foot strikes in seconds, starting with the first matched pair, while the *interval ipsilateral cues* represents the duration in seconds between two ipsilateral cues [20] (Figure 2d). A 360° phase progression corresponds to a full stride. For the stride-based pacing conditions, a virtual cue was used to calculate the relative phase for each foot strike (i.e., paced and unpaced step).

The cumulative relative-phase error was calculated for each window of 50 steps in the synchronization phase for the synchronization-perturbation conditions. This error was defined as the sum of consecutive changes in the relative phase and calculated according to:

(2) $$Cumulative\;relative\;phase\;error=\sum_{step=2:n}(\phi_{step}-\phi_{step-1})$$

in which *ϕ* is the relative phase and *step* is the step for which the relative-phase error is calculated. For all windows with an absolute cumulative relative-phase error below 54°, we determined the standard deviation of the relative phase. If there was no window with an absolute cumulative relative-phase error below 54° [20], or if the lowest standard deviation of the relative phase was greater than 30°, we deemed synchronization to be absent. The number of participants who achieved synchronization in each condition was analyzed using Friedman’s ANOVA and Wilcoxon signed ranks test. If synchronization was achieved, the window with the lowest standard deviation of the relative phase was used for further analysis. The constant error of the normalized cadence was determined as the mean difference between the cadence and the target cadence, such that a positive difference indicates that the cadence is higher than the target. Furthermore, circular statistics [36] was used to calculate the mean (magnitude of the anticipation tendency in auditory-motor coordination) and variability (stability of auditory-motor coordination) of the relative phase (*ϕ*).

#### 2.2.2. Synchronization-Perturbation Task

There were three possible valid responses to a perturbation: a 60° correction towards the pre-perturbation relative phase (typical response), a 120° correction leading to a half-cycle phase shift, and a 300° correction leading to a full-cycle phase shift. We used the criteria as specified in [20] to determine whether a response was valid and to classify the responses as typical, half-cycle phase shift and full-cycle phase shift. If the response to the perturbation was classified as typical, the number of steps needed to correct the perturbation and return to stable auditory-motor coordination was determined according to [20].

#### 2.2.3. Synchronization-Continuation Task

The continuation phase was only analyzed if synchronization was achieved during the synchronization phase of this task, that is, when the absolute cumulative relative-phase error over the final 50 steps was smaller than 54° [20] and the standard deviation of the relative phase was smaller than 30°. To examine how well participants were able to continue moving at the imposed cadence after removal of the cues we defined borders of 0.05 around the targeted normalized cadences, leading to five ranges into which the cadences (per step) during synchronization and continuation were distributed: below 0.85, 0.85 to 0.95, 0.95 to 1.05, 1.05 to 1.15, and above 1.15. The constant error, defined as the mean difference between the cadence and the target cadence, such that a positive constant error indicates a cadence higher than the target, was calculated for the first 100 steps after removal of the cues.

#### 2.2.4. Statistical Analysis

The statistical analysis was performed in IBM SPSS Statistics 25. Results were deemed significant at α < 0.05, and effect sizes were quantified as partial eta squared (*η_p_*^2^). Preferred cadences as assessed at the start and end of each locomotion type experimental block were compared using a two-tailed paired-samples *t*-test, accompanied by the intra-class correlation coefficient (*ICC*) for absolute agreement (with values < 0.5, between 0.5 and 0.75, between 0.75 and 0.9 and > 0.9 signaling poor, moderate, good and excellent agreement, respectively [37]). The mean and standard deviation of the relative phase (*ϕ*), the number of steps needed to correct the perturbation, and the constant error of the normalized cadence during synchronization and continuation were compared between conditions using 2 (pacing type) × 2 (locomotion) × 3 (pacing frequency) repeated-measures ANOVA with Bonferroni-corrected post-hoc analyses of significant effects. With regard to the questionnaire, we compared participants’ ratings on difficulty, perceived performance, comfort, and enjoyment with a 2 (pacing type) × 2 (locomotion) repeated-measures ANOVA.

## 3. Results

The comfortable speeds for walking and running were 5.22 ± 0.48 km/h and 10.47 ± 0.94 km/h, respectively. The corresponding preferred cadence for walking was 113.41 ± 5.95 steps/min (114.21 ± 6.27 steps/min after completing the measurements; *ICC* = 0.79; *t*(15) = −0.79, *p* = 0.44). For running, the preferred cadence was 162.20 ± 8.78 steps/min (164.18 ± 9.99 steps/min after completing the measurements; *ICC* = 0.78; *t*(15) = −1.44, *p* = 0.17).

### 3.1. Synchronization Phase

The number of participants who achieved synchronization differed significantly across conditions (*Χ*^2^(16) = 31.97, *p* = 0.001). Significantly less participants achieved synchronization in the running conditions than in the walking conditions (*T* = 0, *r* = −0.68; Table 2). Furthermore, synchronization was achieved less often for the slow pacing frequency compared to preferred (*T* = 0, *r* = −0.64) and fast (*T* = 6, *r* = −0.50) pacing frequencies (Table 2). Five out of 16 participants achieved synchronization in all conditions, and three out of 16 participants achieved synchronization in (less than) half of the conditions. The other eight participants achieved synchronization in 9 to 11 out of 12 conditions. The participants who synchronized with the pacing signal did so with the normalized cadences shown in Table 2.

Due to the lack of synchronization in at least one condition, 11 participants would be excluded from the planned repeated-measures ANOVA. Given the distribution of invalid conditions in the running conditions with detuning, we instead used a 2 (pacing type) × 2 (locomotion) repeated-measures ANOVA including only the conditions with the preferred pacing frequency, and a 2 (pacing type) × 3 (pacing frequency) repeated-measures ANOVA including only the walking conditions. This led to the inclusion of 13 and 11 participants, respectively.

The constant error of the normalized cadence during synchronization with preferred pacing frequency was 0.001 ± 0.001. There were no significant differences in constant error across locomotion types and pacing types. However, there was a significant effect of frequency on the constant error, *F*(1.20, 11.97) = 56.37, *p* = 0.000, *η_p_*^2^ = 0.85 (Greenhouse-Geisser corrected values are reported, because Mauchly’s test was significant, *p* = 0.007). Post-hoc tests with Bonferroni correction revealed a small, but significantly higher constant error for the slow (0.009 ± 0.005) and fast (0.012 ± 0.001) pacing frequencies compared to the preferred pacing frequency (0.001 ± 0.001; *p* < 0.001).

**Figure 3:**
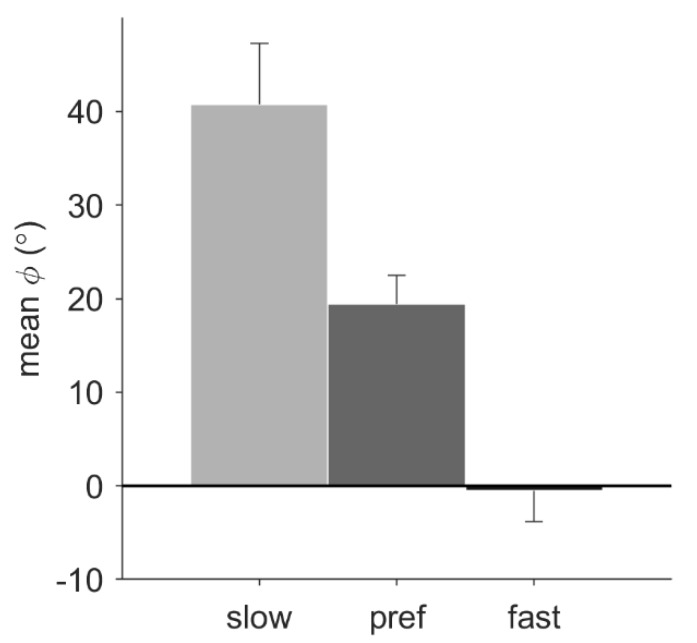
Mean relative phase (*ϕ*) during stationary auditory-motor coordination for walking with the different pacing frequencies (*N* = 11). Error bars represent the standard error of the mean.

For the walking conditions, pacing frequency had a significant effect on the mean relative phase, *F*(1.24, 12.37) = 18.56, *p* = 0.001, *η_p_*^2^ = 0.65. Post-hoc tests with Bonferroni correction revealed that the mean relative phase of slow (38.83° ± 22.27°), preferred (18.93° ± 10.85°), and fast (−0.79° ± 13.09°) pacing-frequency conditions all differed significantly from each other (*p* < 0.05; Figure 3). For the conditions with the preferred pacing frequency, a trend suggested a larger mean relative phase for running (33.81° ± 21.61°) than for walking (21.66° ± 11.18°), *p* = 0.053. No significant main effect of pacing type was found, nor any significant interactions.

**Figure 4:**
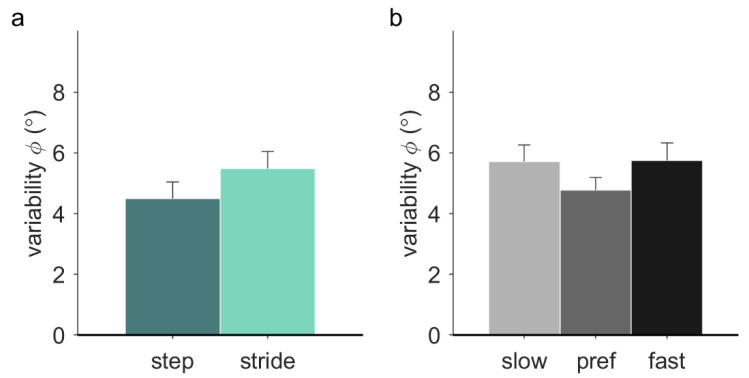
Variability of the relative phase (*ϕ*) during stationary auditory-motor coordination. (**a**) The different pacing types (*N* = 13) (**b**) The different pacing frequencies (*N* = 11). Error bars represent the standard error of the mean.

Variability of the relative phase was significantly higher with stride-based (5.48° ± 2.01°) than step-based pacing (4.50° ± 1.92°), *F*(1, 12) = 8.68, *p* = 0.012, *η_p_*^2^ = 0.42 (Figure 4a). There was a significant effect of frequency on the variability of the relative phase in walking (*F*(2, 20) = 3.75, *p* = 0.042, *η_p_*^2^ = 0.27). Post-hoc tests with Bonferroni correction revealed no significant individual differences between slow (5.72° ± 0.55°), preferred (4.78° ± 0.41°), and fast (5.75° ± 0.58°) pacing frequencies (Figure 4b). No significant effect of locomotion was found, nor any significant interactions.

### 3.2. Perturbation Analysis

**Figure 5:**
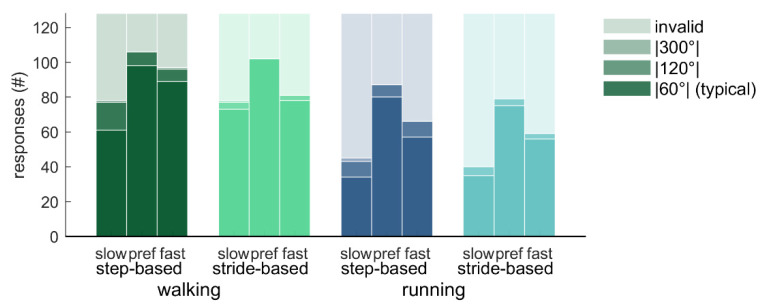
Distribution of response types over the different conditions. The total number of perturbations was 128 (16 participants times eight perturbations per condition).

**Figure 6:**
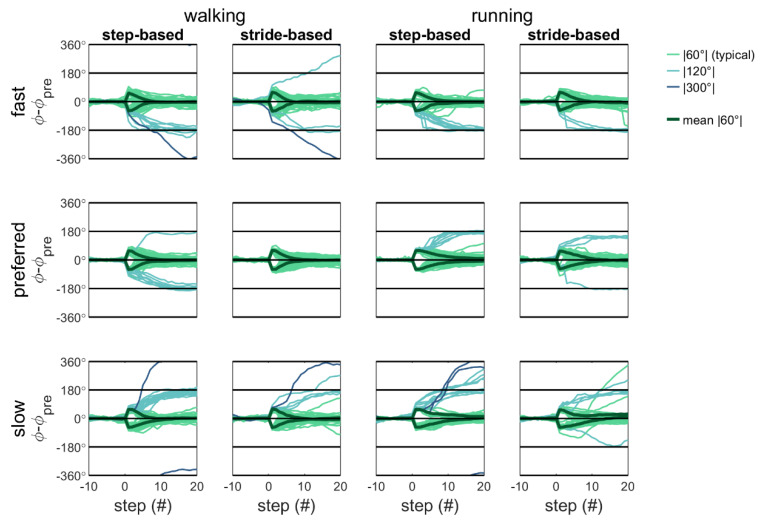
Relative phase for the valid responses to the perturbations with the mean pre-perturbation subtracted. The relative phase for walking is presented on the left side and that for running on the right side. A positive perturbation is a delay in the cues and a negative perturbation is an advance in the cues.

How often the different responses to the perturbations occurred varied across conditions (Figure 5 and Figure 6). Friedman’s ANOVA indicated that there were significant differences in the number of typical responses (|60°|) across conditions (*Χ*^2^(11) = 63.95, *p* < 0.001; Figure 5). Wilcoxon tests with Bonferroni correction were used to make specific comparisons. Participants had less typical responses for running (3.51 ± 1.98; out of 8 perturbations) than for walking (5.22 ± 2.11; *T* = 0, *r* = 0.88), and less typical responses for slow pacing frequency conditions (3.17 ± 2.11) than for preferred frequency conditions (5.55 ± 1.95; *T* = 1.5, *r* = 0.86). The number of typical responses did not differ between step-based (4.36 ± 1.96) and stride-based (4.36 ± 2.15) pacing conditions. Similar but opposite differences were found for the number of invalid responses (*Χ*^2^(11) = 63.88, *p* < 0.001; *T* = 0, *r* = 0.88; *T* = 1.5, *r* = 0.86, respectively; Figure 5).

Qualitative inspection of Figure 5 and Figure 6 reveals that with detuning, atypical (|120°| and |300°|) responses occur more often and with step responses in the direction of the preferred cadence. That is, participants predominantly adopt a slower-step response to correct for a phase-advance perturbation (−60°) with fast pacing frequency (yielding −120° and −300° adjustments) and a faster-step response to correct a phase-delay perturbation (+60°) with slow pacing frequency (yielding +120° and +300° adjustments [4]).

**Figure 7:**
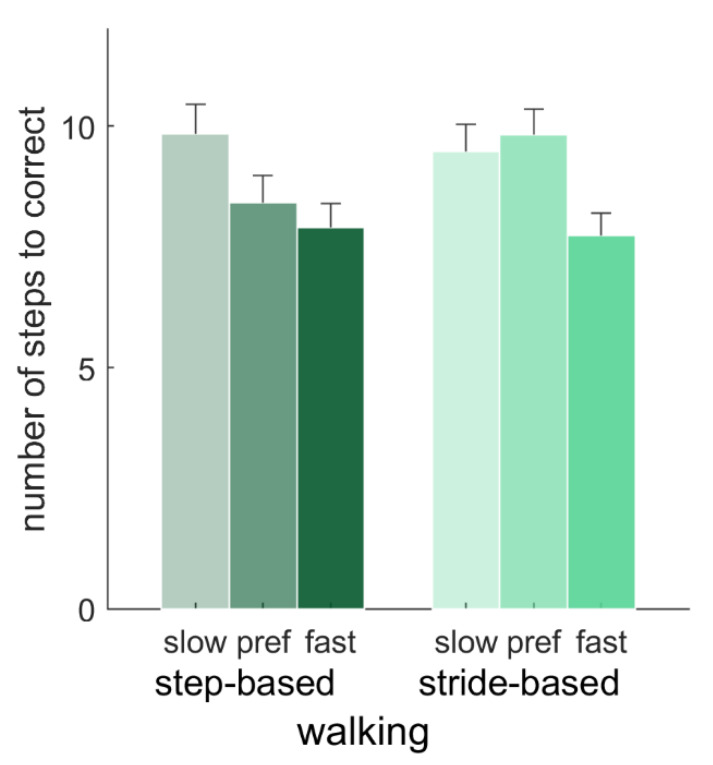
Average number of steps needed to correct after perturbations in walking (*N* = 12). Error bars represent the standard error of the mean.

For typical responses, the number of steps needed to correct for a perturbation were compared with a 2 (pacing type) × 2 (locomotion) repeated-measures ANOVA (*N* = 15; preferred frequency conditions only), and with a 2 (pacing type) × 3 (pacing frequency) repeated-measures ANOVA (*N* = 12; walking conditions only). There were no significant main effects of pacing type, locomotion or the pacing type x locomotion interaction (*p* > 0.05) on the number of steps needed to correct for a perturbation with the preferred pacing frequency. For the walking conditions, there was a significant effect of frequency on the number of steps needed to correct, *F*(2, 22) = 8.06, *p* = 0.002, *η_p_*^2^ = 0.42. Post-hoc tests showed that this number was significantly higher for slow (9.66 ± 1.84) than fast (7.81 ± 1.48) pacing frequencies (*p* = 0.011), but neither of those differed significantly from the number of steps needed for the preferred pacing frequency (9.12 ± 1.77). Furthermore, there was a significant interaction of pacing type and pacing frequency, *F*(2, 22) = 4.77, *p* = 0.019, *η_p_*^2^ = 0.30 (Figure 7). Post-hoc tests with Bonferroni correction revealed that for the preferred pacing frequency, the number of steps needed to correct for a perturbation was significantly lower with step-based (8.41 ± 1.96) than with stride-based (9.83 ± 1.80) pacing, *p* = 0.003, in the absence of significant differences between pacing types for slow and fast pacing frequencies.

### 3.3. Continuation Analysis

**Figure 8:**
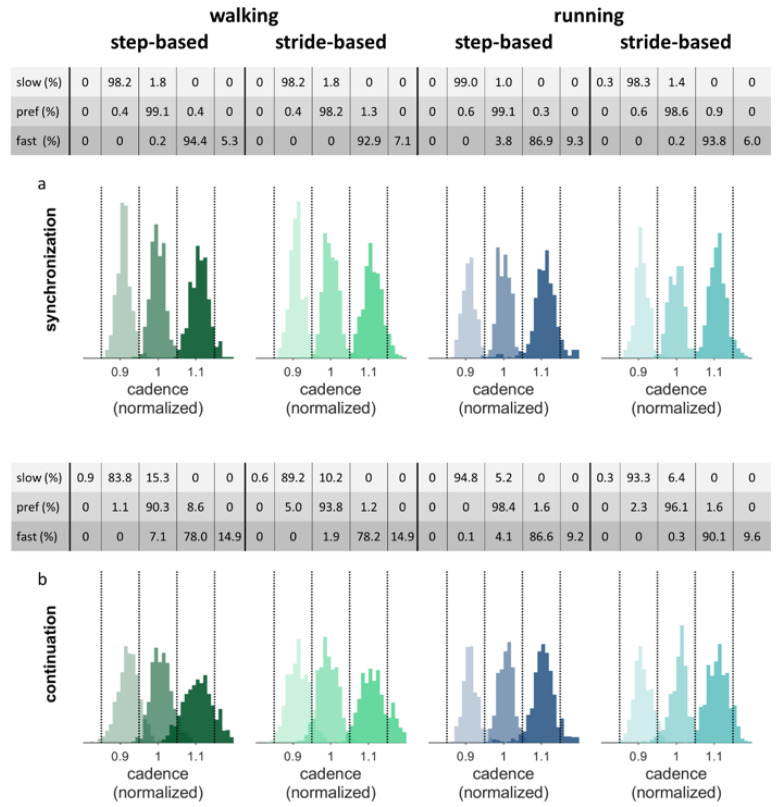
Histograms of the normalized cadence. (**a**) Synchronization phase. (**b**) Continuation phase. Vertical dotted lines represent the borders of the ranges around the slow (0.9), preferred (1.0) and fast (1.1) frequencies. The percentages of cadences falling in each frequency range are displayed above the figure for the three pacing frequencies separately. The data displayed belongs to the nine participants included in the constant error analysis for walking. As some of these participants did not synchronize in all conditions, displayed histograms were based on *N* = 7 for running with preferred step-based, and slow and preferred stride-based pacing conditions and *N* = 6 for the running with slow step-based pacing conditions.

If stable auditory-motor coordination was not achieved during the final 50 steps of the synchronization phase, participants were excluded from the analysis of the continuation phase. Figure 8 shows the histograms of the normalized cadence data separately for the synchronization and continuation parts of the conditions for the nine participants who achieved synchronization in all walking trials. The figure shows that the cadence, by and large, remains within the correct range during continuation. The tables above the separate figures show the percentage of steps with the cadence in the corresponding range per condition.

The constant error between the target cadence and the performed cadences during continuation were compared with a 2 (pacing type) × 2 (locomotion) repeated-measures ANOVA (*N* = 9; preferred frequency conditions only), and with a 2 (pacing type) × 3 (pacing frequency) repeated-measures ANOVA (*N* = 9; walking conditions only). In line with the qualitative inspection of Figure 8, no significant main or interaction effects of pacing type and locomotion were found in the preferred frequency conditions (0.002 ± 0.009). In walking, there was a significant effect of pacing frequency on the constant error, *F*(2, 16) = 3.81, *p* = 0.044, *η*_p_^2^ = 0.32. Post-hoc tests with Bonferroni correction revealed a higher constant error for the slow pacing frequency (0.019 ± 0.018) compared to the preferred frequency (0.001 ± 0.015, *p* < 0.05). Figure 8 indeed shows that the histograms for the slow pacing frequency are shifted somewhat to the right, that is, in the direction of one’s preferred cadence. No significant interactions were found.

### 3.4. Questionnaire

**Figure 9:**
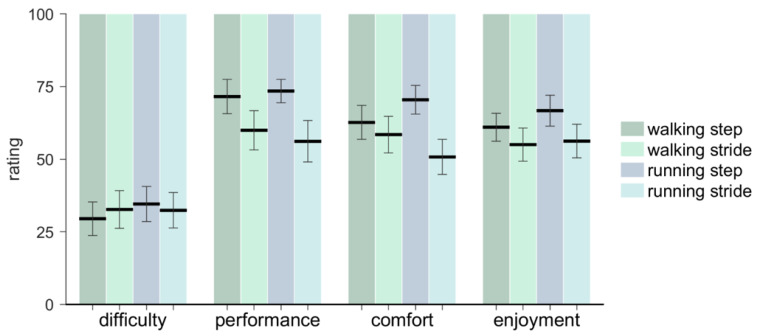
The mean and standard error of the ratings for perceived difficulty, performance, comfort and enjoyment for walking and running with step-based and stride-based pacing. On the scale, a low rating means low perceived difficulty/performance/comfort/enjoyment.

The results of the questionnaire are presented in Figure 9. Individual participant ratings are available in the Supplementary Materials. Participants rated their synchronization performance to be better for step-based (73.07 ± 17.00) than for stride-based pacing (59.37 ± 24.67; *F*(1, 14) = 6.06, *p* = 0.027, *η*_p_^2^ = 0.30). They further rated comfort higher for step-based (66.47 ± 18.71) than for stride-based (55.13 ± 22.73) pacing (*F*(1, 14) = 4.97, *p* = 0.043, *η*_p_^2^ = 0.26). Difficulty and enjoyment rates did not differ between step-based and stride-based pacing (*p* > 0.05). No significant differences in the ratings between walking and running were found, nor any significant interactions.

## 4. Discussion

In this study, we compared, in sixteen experienced runners, the effectiveness of step-based pacing and stride-based pacing for modulating and internalizing cadence in walking and running, for pacing frequencies slower than, equal to, and faster than the preferred cadence of the participants. We expected synchronization to be achieved more often with step-based (stronger coupling) than with stride-based pacing. However, no evidence was found for this expectation. If synchronization was achieved, we further expected auditory-motor coordination to be more stable for step-based pacing than for stride-based pacing for both walking and running. Auditory-motor coordination was indeed more stable with step-based pacing, as evidenced by significantly lower variability in the relative phase between steps and cues. Furthermore, fewer steps were needed to correct for a perturbation with step-based pacing than stride-based pacing, but only with preferred pacing frequency in walking; no significant main effect of pacing type was found. These findings indicate that stronger coupling (i.e., pacing steps) leads to more stable auditory-motor coordination, as was found in a previous study reporting superior auditory-motor synchronization for step-based pacing in walking [4]. With regard to the participants’ preference, ratings suggested that step-based pacing was perceived to be more comfortable for both walking and running and led to a higher perceived performance than stride-based pacing. These subjective ratings in favor of step-based pacing are consistent with the objective findings showing superior coordinative stability of step-based pacing. In view of the superior auditory-motor coordination and better subjective ratings by the participants, we would advise walkers and runners (and their therapists and trainers) to opt for step-based pacing rather than stride-based pacing for the purpose of cadence modulation.

In the present study, we also systematically manipulated detuning, and expected that participants would achieve synchronization more often with pacing matching one’s preferred frequency (no detuning) than with slower-than-preferred and faster-than-preferred pacing frequencies (detuning). More participants were excluded in conditions with detuning due to the absence of synchronization, and there were less typical responses to perturbations, but only for conditions with a slower-than-preferred pacing frequency. This suggests that synchronizing to a slower-than-preferred pacing signal is more difficult than synchronizing to a pacing signal that matches one’s preferred cadence, but synchronizing to a faster-than-preferred pacing frequency is not. If synchronization was achieved, we expected auditory-motor coordination to be most stable for the preferred pacing frequency conditions (no detuning). Coordination was indeed more stable (lower variability of the relative phase) without detuning, which is consistent with the literature [13]. Furthermore, the correction after perturbations was slower with slow than fast pacing frequency, but neither was significantly different from the preferred frequency. These combined results suggest that the negative effects of detuning on the stability of the auditory-motor coordination are more pronounced for slow than for fast pacing. The goal of the present study was to modulate cadence with the practical application of reducing injury. The increase in cadence of 10% recommended for that purpose [6,7] seems to be feasible with auditory pacing, with little loss of stability of auditory-motor coordination and, as will be discussed below, with internalization of this faster cadence after a relatively brief period of pacing.

We expected a detuning-related change of the cadence in the direction of the preferred cadence. During the synchronization phase, the constant error was indeed positive for the slow pacing frequency, indicating that the cadence tended to be somewhat faster than the slow pacing frequency, but it was also positive with fast pacing. In addition, detuning did have the expected effect on the lead-lag relationship between steps and pacing cues, with a larger phase lead with slow and a smaller phase lead (in some cases a phase lag) for fast pacing [13], as evidenced respectively by a significantly higher and lower mean relative phase compared to the preferred pacing frequency, confirming that the inherently faster oscillator tends to lead [12,13]. Furthermore, the participants who achieved synchronization did remain at the target cadence after the removal of the pacing signal. We expected the constant error during continuation to be larger (positive) for the slow and smaller (negative) for the fast pacing frequency than for the preferred pacing frequency, but constant error, albeit low, was only significantly larger for the slow compared to the preferred pacing frequency, indicating a slightly worse internalization of the slower-than-preferred target frequency; no significant difference in constant error between the fast and preferred pacing was found. Participants were able to continue at the target cadence for 100 steps after removal of the pacing signal, provided that footfalls were synchronized to the pacing signal in the synchronization phase, since unsynchronized participants were excluded from this analysis.

We did not expect synchronization and stability of auditory-motor coordination to be different for walking and running. However, there were more conditions in which synchronization was not achieved, and more invalid responses to perturbations for running compared to walking, suggesting that synchronization was more difficult to achieve for running, especially at slow pacing frequencies. A plausible explanation for this difference is not readily apparent, but it may be the case that running, in particular on a treadmill, is a less automated activity than walking, and therefore more susceptible when combined with another attention-demanding task like synchronizing footfalls to an auditory metronome. Previous studies have shown that paced walking is more attention demanding than unpaced walking [38,39], and the same probably holds for running, but perhaps in a stronger manner. Note, however, that if synchronization was achieved, auditory-motor coordination was not significantly different for walking and running.

As already intimated, our findings may have practical implications for applying auditory pacing for modulating and internalizing cadence in the fields of rehabilitation and sports. However, before generalizing the current results to practice, a couple of limitations should be taken into account. Firstly, three of the participants did not achieve synchronization in (more than) half of the conditions. This could indicate that these participants had difficulty with auditory-motor synchronization in general [40]. For such poor synchronizers, auditory pacing will not be an effective method for modulating cadence. Furthermore, in this study participants walked and ran on a treadmill. Treadmill locomotion differs somewhat from over-ground locomotion since the speed must be kept constant to avoid falling off the treadmill, implying that participants needed to tightly control their speed in light of this constraint [41]. Consequently, modulating cadence through pacing also implied modulating step lengths (e.g., an increase in cadence is accompanied by a decrease in step length to maintain the same speed). In over-ground locomotion, in contrast, speed and step length may be adjusted independently of one another when modulating cadence. Increasing cadence over-ground through pacing may thus result in increased step lengths (and hence speed), which may be problematic in that greater step lengths are associated with injury risk in running [8].

Given the aforementioned limitations, our recommendations for future research are to use a poor ability to synchronize as an exclusion criterion, as the poor synchronizers were excluded from most analyses in this study. In addition, we recommend future studies designed to extend our findings to over-ground running to pace cadence in relation to the actual speed in over-ground running, that is, if the use of auditory pacing is intended for modulating cadence with the aim of reducing injury risk. In relation to the latter recommendation, it is important to note that the energetically optimal cadence is faster than the preferred cadence in both treadmill [42,43] and over-ground [44] running, and can be determined as the cadence corresponding to the lowest heart rate for each speed [42,43,44]. We thus recommend future research to modulate cadence towards this energetically optimal cadence, as it may reduce both energy cost and injury risk.

## Figures and Tables

**Table 1:** Overview of the different conditions and the order of these conditions.

Locomotion	Task	Pacing Type	Pacing Frequency
counterbalanced	fixed order	counterbalanced	random order
running	synchronization-perturbation	step-based	Slow
			Preferred
walking	synchronization-continuation	stride-based	
			Fast

**Table 2:** Normalized cadence during stationary auditory-motor coordination (mean ± standard deviation (sd)) and the number of participants *N* who did (not) achieve synchronization.

		Step-Based			Stride-Based		
		**Slow**	**Preferred**	**Fast**	**Slow**	**Preferred**	**Fast**
**Walking**	**Mean ± sd**	0.91 ± 0.009	1.00 ± 0.002	1.11 ± 0.002	0.91 ± 0.004	1.00 ± 0.001	1.11 ± 0.003
	***N* (Missing)**	12 (4)	15 (1)	15 (1)	14 (2)	15 (1)	14 (2)
**Running**	**Mean ± sd**	0.91 ± 0.004	1.00 ± 0.002	1.11 ± 0.001	0.91 ± 0.003	1.00 ± 0.003	1.11 ± 0.002
	***N* (Missing)**	9 (7)	14 (2)	13 (3)	9 (7)	15 (1)	12 (4)

## Supplementary Materials

The following is available online at https://zenodo.org/record/3861849#.Xs8d48B5uUk, The dataset for this study, comprising preprocessed series, calculated series and event streams per participant and condition.
